# Therapeutic Potential of Stylissatin A and Related Cyclic Peptides From Marine Sponges

**DOI:** 10.1002/psc.70045

**Published:** 2025-07-24

**Authors:** Aaqib Ullah, Farzana Shaheen, Uzma Salar, Andreas G. Tzakos, Ioannis P. Gerothanassis

**Affiliations:** ^1^ Third World Center for Science and Technology, H.E.J Research Institute of Chemistry, International Center for Chemical and Biological Sciences University of Karachi Karachi Pakistan; ^2^ Section of Organic Chemistry and Biochemistry, Department of Chemistry University of Ioannina Ioannina Greece; ^3^ Dr. Panjwani Center for Molecular Medicine and Drug Research, International Center for Chemical and Biological Sciences University of Karachi Karachi Pakistan

**Keywords:** anticancer, anti‐inflammatory, anti‐obesity, marine sponges, *Stylissa massa*, stylissatin A (SA)

## Abstract

Marine sponges are sessile invertebrates found in moderate, arctic, and tropical regions, serving as a valuable reservoir of bioactive compounds, particularly Pro‐rich peptides. Among these, cyclic peptides have attracted significant interest due to their diverse therapeutic properties. One notable example is Stylissatin A (SA), a Pro‐rich cyclic peptide reported from the marine sponge *Stylissa massa*. SA and its analogues have shown promising biological activities, including anti‐inflammatory, anticancer, and anti‐obesity effects. Despite the vast potential of marine‐derived peptides, only a small number have progressed to the pharmaceutical market. Cyclic peptides like SA offer unique opportunities for molecular modifications and total synthesis, enabling the enhancement of potency, improvement of physicochemical properties, and optimization of synthetic yields. This review highlights the synthetic strategies developed for the total synthesis of SA, explores its structural features and related analogues, and discusses their therapeutic potential, underscoring the promise of SA‐based scaffolds as novel peptide‐based drug candidates.

## Graphical abstract

Synthesis and biologicals activities of the natural cyclic peptide stylissatin A and its various analogues arereported. The findings suggest that specific amino acid residues such as Ile at position 2, Pro at 4 and 6, and Tyr at position 1 are key contributors to the peptide's therapeutic potential.

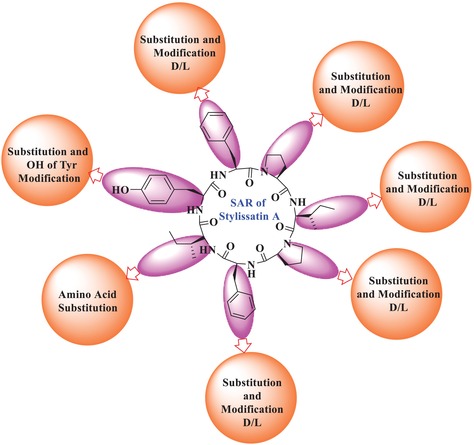

## 1 Introduction

Marine animals are rich sources of bioactive natural products with unique structural identities [1, 2]. Many researchers have been focusing on discovering marine‐derived potent drug candidates. Among these, sponges have been extensively considered, and various bioactive natural molecules have been isolated in the last two decades [3, 4, 5]. There are about 10,000 sponges found commonly crosswise in the sea, and numerous pharmaceutically active natural cyclic peptides have been recognized from these sponges, and many of these peptides and their analogues are in clinical developmental phases [6, 7, 8].

Most peptides reported from marine sponges are identified as Pro‐rich cyclic peptides and depsipeptides [9]. It has also been reported that sponge genera like *Stylissa*, *Stylotella*, *Phakellia*, *Axinella*, and *Hymeniacidon* are rich sources of cyclic peptides containing 7–12 amino acids like wainunuamide [10], phakellistatins [11], axinastatin [12], stylissamides [13], axinellins [14], stylissatin A‐D [15], solomonamides A and B [16], perthamide C, perthamide D [17], and stylisins [18] with significant structural stability and biological activities. Compared to their linear counterparts, cyclic peptides exhibit enhanced resistance to proteolytic degradation and maintain greater stability within the gastrointestinal tract. Their cyclic conformation confers increased structural rigidity, metabolic stability, and target selectivity, allowing them to effectively engage specific biological sites. These advantageous properties make cyclic peptides highly promising candidates for therapeutic applications, offering a balance of safety, stability, and efficacy as drug molecules [19, 20]. Some of the cyclic peptides, such as halipeptin A [21], cyclomarins A [9], sclerotiotide L, stylissamide X [22], perthamides C [23], solomonamide A [24], and stylissatin A (SA), showed anti‐inflammatory activities evaluated via various bioassays [25].

**FIGURE 1:**
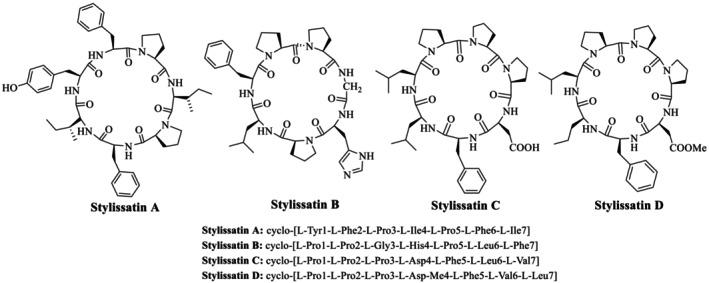
Structures of stylissatin A–D, natural cyclic peptides from *Stylissa massa* marine sponge, and their amino acid sequences.

Stylissatins A–D are cyclic peptides isolated from *Stylissa massa* (Figure 1). All of these natural peptides characteristically consist of seven to eight amino acid residues and contain two Pro‐rich residues that are arranged in sequence in stylissatin B–D. Notably, these Pro residues exhibit *cis*‐*trans* isomerization, making them interconvertible within the sequence. This is the first reported instance of a natural cyclopeptide containing two sequential proline units demonstrating such *cis*‐*trans* interconversion, highlighting a unique structural feature in naturally occurring cyclic peptides [26]. Among the four cyclic peptides, SA gained significant therapeutic importance. SA is described to show an inhibition against nitric oxide (NO) production in LPS‐stimulated murine RAW264.7 macrophage cells with very little cell toxicity [15], while other cyclic peptides, stylissatin B, C, and D, were found to show less inhibitory effect toward NO production [12]. In addition, stylissatin B was also reported to exhibit inhibition toward a panel of tumor cell lines, that is, HCT‐116, HepG2, BGC‐823, NCI‐H1650, A2780, and MCF‐7 [27].

This mini‐review summarizes the total syntheses of SA reported to date through various synthetic routes, along with the documented therapeutic potential of SA and its related analogues.

## 2 Synthesis of SA

### 2.1 Solid‐Phase Total Synthesis of SA

**SCHEME 1:**
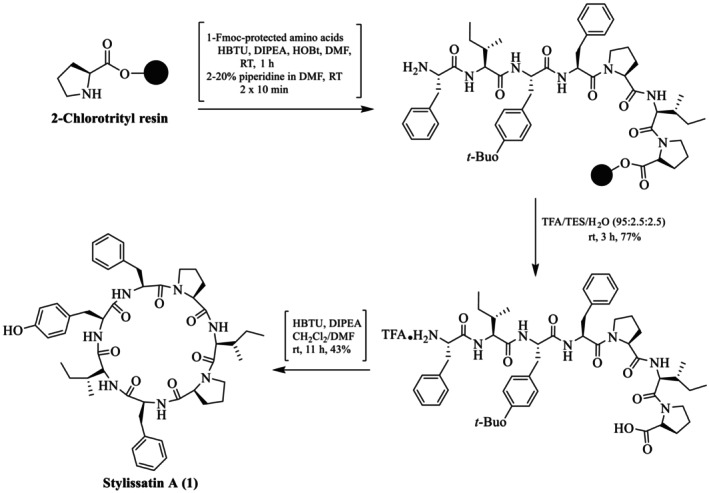
Solid phase peptide synthesis (SPPS) of SA (**1**) by Akindele et al. [28].

Akindele et al. reported the first total synthesis of SA (**1**) natural cyclic peptide through solid phase (SP) by employing 2‐chlorotrityl resin as a solid support (Scheme 1) [28]. First, a Pro residue was coupled to the resin to reduce epimerization in the macrolactamization step. The cyclization site between Phe^3^ (N‐terminus) and Pro^4^ (C‐terminus) was selected to avoid steric hindrance at the position of cyclization. HBTU, HOBt, and *N*, *N*‐diisopropylethylamine (DIEA) were used in anhydrous DMF as coupling reagents at ambient temperature. The Fmoc protective group was removed by 20% piperidine in DMF. The desired linear amino acid sequence heptapeptide and *tert*‐butyl group were cleaved simultaneously from the resin to obtain the crude peptide, which was further purified by reverse phase HPLC (Develosil ODS‐HG‐5, 75%–85% aq. MeOH) and obtained at 77% yield. In the last step, cyclization was performed by HBTU in CH_2_Cl_2_/DMF (5/1) and dropwise addition of DIPEA in CH_2_Cl_2_, giving a 43% yield. Finally, the cyclic stylissatin A was purified by a reversed‐phase HPLC (Develosil ODS‐HG‐5, 75%–85% aq MeOH). The structure was fully characterized by mass spectrometry and NMR spectroscopy, which matched that of the natural product.

**SCHEME 2:**
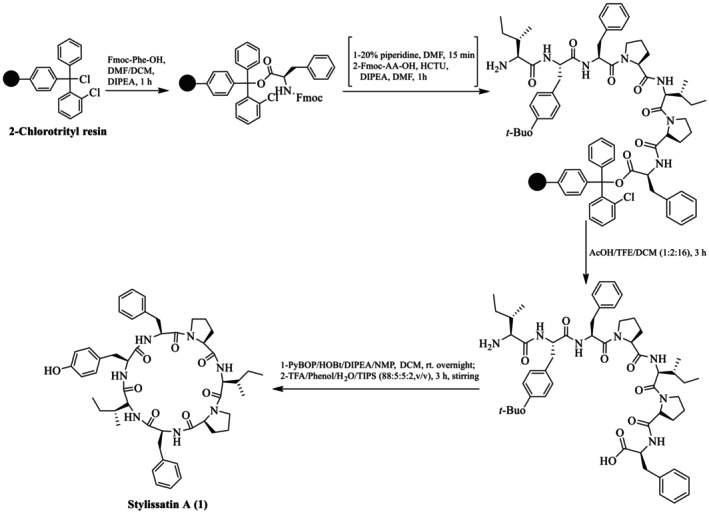
SPPS of SA reported by Li et al. [29].

Li et al. also reported the total synthesis of SA (**1**) through SPPS by using 2‐chlorotrityl chloride resin [29]. Cyclization was performed between the terminus Ile^2^ and Phe^3^ amino acids of linear peptide amino acid residues (Scheme 2). The first coupling was started by loading Phe on 2‐chlorotrityl chloride resin using DIEA in DMF/DCM for 3 h, followed by repeating the same procedure (Fmoc protocol) to synthesize the desired sequence of linear heptapeptide precursor. Then, the linear peptide was cleaved from the resin by using a TFA cocktail, that is, AcOH/TFE/DCM (1:2:16), stirring for 3 h at room temperature. The cyclization was performed via solution phase by using PyBOP/HOBt/DIPEA/NMP as coupling reagents in DCM (to reduce byproducts) to obtain the target molecule. The crude peptide was purified on reverse phase preparative HPLC (recycling) using a gradient solvent system, that is, acetonitrile in water, 90%–20%, with a retention time of 27 min and 98% purity for stylissatin A. The structure of the desired cyclic peptide SA (**1**) was characterized through ESI‐MS, HR‐QTOF‐MS, ^1^H‐NMR, and ^13^C‐NMR spectroscopies.

**FIGURE 2:**
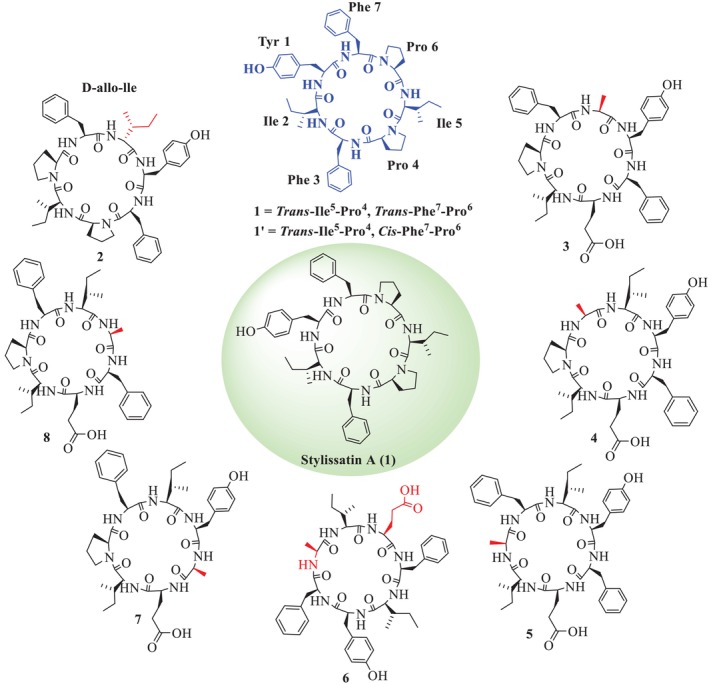
SPPS of analogues of SA (**1**) by Shaheen et al. [30].

**SCHEME 3:**
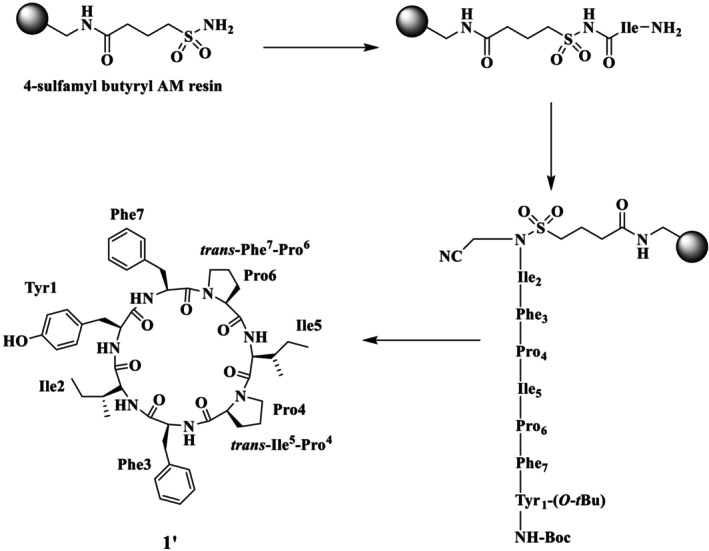
SPPS of SA (**1′**) through route A by Shaheen et al. [30].

Shaheen et al. also reported the total synthesis of the natural product SA (**1**) and its Pro rotamer (**1′**), epimer (**1″**), and its analogues (**2–8**), as illustrated in Figure 2, through the SP via Kenner's sulfonamide safety‐catch linker strategy (solid support) [30]. Furthermore, the same group reported two synthetic routes for the synthesis of **1**, using different sites for macrocyclization. In route A, cyclization was performed between Tyr^1^ and Ile^2^ and resulted in **1′** as the major product. A comparison of the NMR chemical shifts of the natural product **1** and the synthetic peptide **1′** in DMSO‐*d*_*6*_ was performed. In the natural product **1**, a *trans*, *cis* isomeric configuration at the Ile^5^‐Pro^4^ and Phe^7^‐Pro^6^ peptide bonds, respectively, was determined from the analysis of the ^13^C‐NMR chemical shift differences between the C*β* and C*γ* atoms of the Pro^4^ and Pro^6^ residues more than 6 ppm (Scheme 3). In contrast, in the synthetic peptide **1′**, the *trans*, *trans* isomerism was confirmed by ^13^C‐NMR chemical shift differences Cβ‐Cγ of Pro^4^ and Pro^6^ (i.e., 4.5 and 4.8 ppm, respectively). This configuration was further determined by NOESY, which showed clear cross peaks between Ile^5^‐Hα/Pro^4^‐Hδ_A_ and Pro^4^‐Hδ_B_ as well as between Phe^7^‐Hα/Pro^6^‐Hδ_A_ and Pro^6^‐Hδ_B_ that further supported the *trans* isomerism of all proline peptidic linkages of **1′**. It was concluded that as the isoleucine residue was involved in the macrocyclization step, the chemical shift differences between **1** and **1′** may be due to epimerization at Ile^2^ during macrocyclization.

**SCHEME 4:**
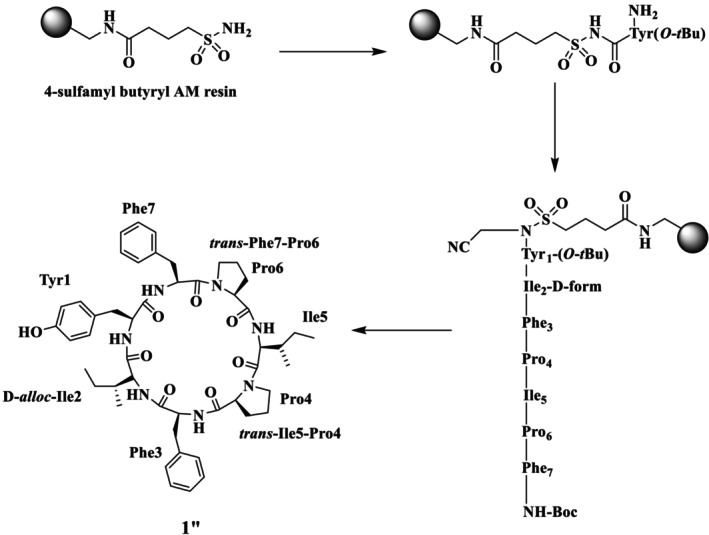
SPPS of SA (**1″**) through route B reported by Shaheen et al. [30].

The synthesis of SA was also performed through route B to exclude the possibility of epimerization using Tyr^1^ and Phe^7^ sites for cyclization along with the replacement of L‐Ile^2^ with D‐*allo*‐Ile^2^ to ensure the full epimerization at this position (Scheme 4). Detailed NMR studies revealed that **1′′** also adopts *trans*‐Ile^5^‐Pro^4^ and *trans*‐Phe^7^‐Pro^6^ isomers. Specifically, ^13^C‐NMR chemical shift differences of Pro^4^ and Pro^6^ Δδ C*ß*‐C*γ* are 4.6 and 4.2 ppm, respectively. The prominent NOESY cross peaks between Ile^5^‐Hα/Pro^4^‐Hδ_A_ and Pro^4^‐Hδ_B_ as well as between Phe^7^‐Hα/Pro^6^‐Hδ_A_ and Pro^6^‐Hδ_B_ confirmed the *trans* configuration of both Pro^4^ and Pro^6^ residues. Although both **1′** and **1′′** contain *trans* proline rotamers, they were distinct species, and this confirms that **1′** is in fact a proline rotamer of natural stylissatin A (**1**) rather than an epimer at the Ile^2^ residue.

**SCHEME 5:**
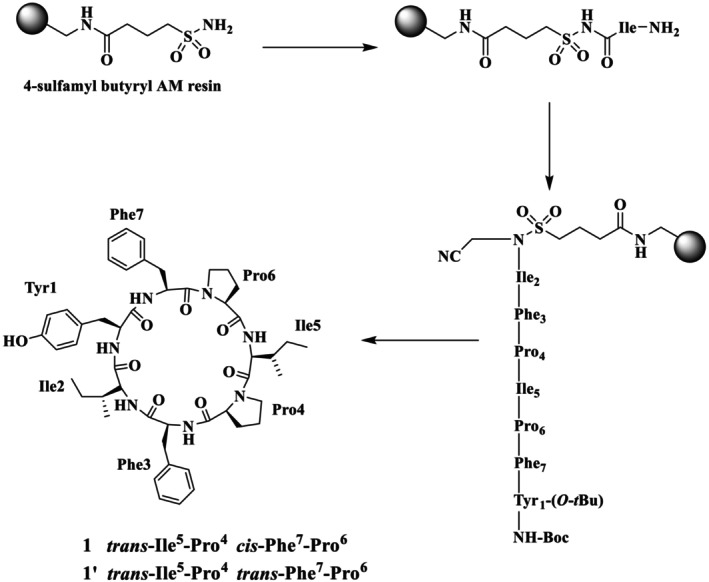
SPPS of SA (**1** and **1′**) through rout B by Shaheen et al. [30].

Shaheen et al. [30] also performed the synthesis with L‐Ile^2^ to explore whether the site of cyclization affects the isomeric population (Scheme 5). Despite the different position of macrocyclization, the synthesis once again led to the *trans*, *trans* proline isomer **1′** as the major product and a small amount of a second product that was isolated and spectroscopically characterized as identical to the *trans*, *cis* natural product isomer **1**. The ^13^C‐NMR chemical shift differences of C*β*‐C*γ* of Pro^4^ and Pro^6^ (i.e., 4.7 and 7.9 ppm) of synthetic SA **1** matched the natural stylissatin A. Further, *cis* isomerism was also confirmed by 2D‐NMR (ROESY) cross peaks between Pro^6^‐Hα and Phe^7^‐Hα for the Phe^7^‐Pro^6^ peptide bond. SA **1**, its proline amide rotamer **1′**, and its epimer **1″** were also found to have different retention times under similar HPLC conditions.

HPLC analysis of the synthetic **1** proline amide isomer **1′** and epimer **1″** was purified using a C4 reversed‐phase analytical column with a mobile phase of 85% MeOH/H_2_O. All analogues were purified through preparative RP HPLC using a Jaigel ODS‐MAT 80 (C18) column using elution with acetonitrile/water (60:40) in 0.08% TFA. The authors further reported that the position of macrocyclization influences the proline isomeric population of synthetic stylissatin A. SA **1** was also found to have similar activity to that reported in the literature.

### 2.2 Solution‐Phase Total Synthesis of SA

**SCHEME 6:**
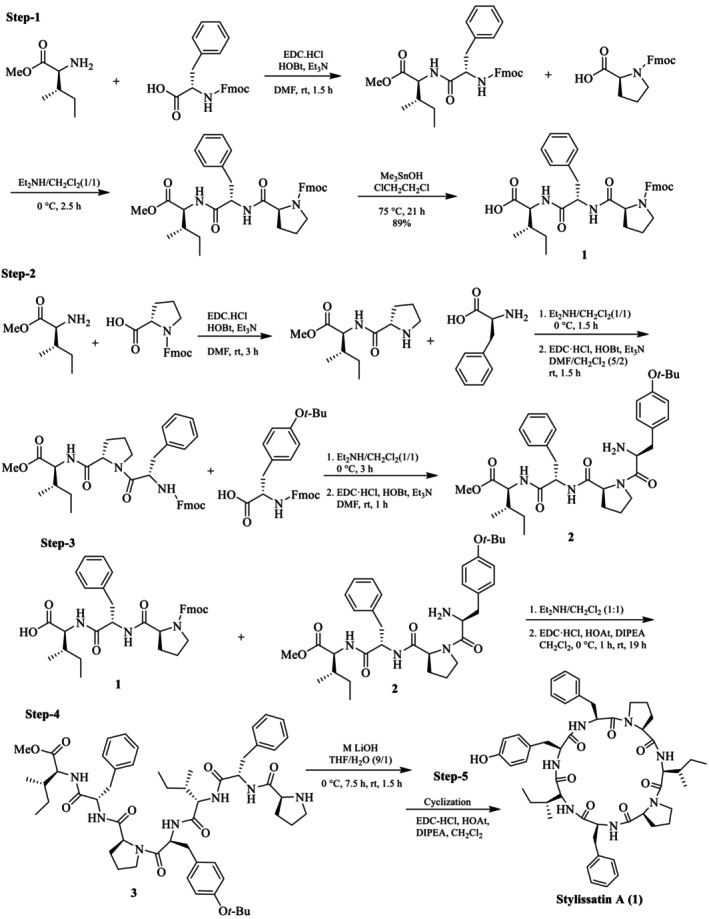
Solution phase synthesis of SA by Akindele et al. [28].

Akindele et al. also reported the solution‐phase synthesis of SA (**1**) [28]. All the desired amino acid residues were coupled by using EDC/HCl and HOBt. Fmoc protecting groups were removed by using 20% piperidine in DMF, and methyl ester protecting groups were cleaved with AlCl_3_/*N*,*N*‐dimethylaniline (9/15) (Scheme 6). Finally, the *tert*‐butyl groups were cleaved from the cyclized peptides by TFA/H_2_O (95:5) to obtain SA (**1**) and its D‐alloc‐Ile.

SA and its analogues have been synthesized using conventional peptide synthesis methods such as SPPS and LPPS. However, due to the significance of peptides as pharmaceutically active candidates, there is a need to develop novel methodologies that can reduce the high cost, number of reaction steps, and toxic waste, while improving purity. Several advanced techniques have been developed to replace traditional peptide synthesis methods. Among these, microwave‐assisted SPPS is commonly employed to reduce reaction time and improve coupling efficiency, yield, and purity. For large‐scale production, automated synthesizers can also be used for high‐throughput peptide synthesis, minimizing labor requirements and offering suitability for industrial applications. Additionally, ultrasound‐assisted solid‐phase synthesis can be utilized to lower costs and hazardous solvent waste by reducing the number of washing steps. Peptides synthesized using this method have also shown good yields and high purity [31, 32, 33].

Recently, green synthetic approaches have been applied to traditional peptide synthesis methods to reduce the use of hazardous chemicals, lower solvent consumption, minimize waste, and enhance coupling efficiency. Green peptide synthesis includes the replacement of toxic solvents such as DMF with ethyl acetate or green ethers, and DCM with MeTHF or ethyl acetate. Additionally, safer and more efficient coupling reagents like Oxyma Pure or COMU are used instead of HBTU or HATU. However, some green solvents are not yet fully compatible with peptide synthesis, and certain green reagents remain expensive or are not widely available [34, 35].

## 3 Therapeutic Applications of SA and Its Related Peptides

To date, several research groups have reported the synthesis and therapeutic potential of SA and its analogues, highlighting anti‐inflammatory effects through the inhibition of nitric oxide (NO) production, reactive oxygen species (ROS), interleukin‐2 (IL‐2) release, and T‐cell proliferation. Additionally, some studies have reported its ability to inhibit adipocyte differentiation and fat accumulation in 3T3‐L1 cells [15, 25, 28, 30, 31]. Herein, the structure–activity relationship (SAR) studies of SA and its structurally related analogues are presented.

### 3.1 Structure–Activity Relationship of SA and Its Related Peptides

SA from the Papua New Guinean marine sponge showed a good inhibition toward NO production in LPS‐stimulated murine macrophage RAW264.7 cells with an IC_50_ of 87 μM [25]. Akindele et al. reported that the synthetic SA peptide showed a similar NO inhibitory activity with no cytotoxicity (EC_50_ = 73 and 87 μM for the synthetic and natural product IC_50_, respectively) [28]. While the D‐allo‐Ile^5^ epimer was reported as inactive, a *tert*‐butyl ether analogue was shown to be about six times more active than SA (EC_50_ = 12 μM).

Further, Shaheen et al. also reported the total synthesis of SA (**1**) and its Pro isomer (**1′**), epimer (**1″**), and its analogues (**2–8**), as illustrated in Figure 2 [30]. The synthetic SA **1** and its *trans*, *trans* isomeric **1′** potently inhibited the NO production. All analogues also demonstrated significant inhibition of reactive oxygen species (ROS) in whole blood and neutrophils. Notably, analogue **3**, featuring an Ala residue at position 2 in the natural product SA, was identified as a particularly potent inhibitor of IL‐2 release and T‐cell proliferation, with IC_50_ values of 6.0 ± 0.9 μM for IL‐2 and 10.9 ± 3.0 μM for T‐cell proliferation (Table 1).

**TABLE 1:** Biological activities of synthetic SA (**1**), Pro isomers of SA (**1′**), (**1″**), and its alanine screening analogues of SA.

Analogues	NO inhibition (μM)	IL‐2 inhibition (μM)	T‐cell proliferation (μM)
**1**	63.0 ± 5.4	—	—
**1′**	60 ± 4.5	—	—
**1″**	69.7 ± 2.3	—	—
**3**	—	6.0 ± 0.9	10.9 ± 3.0
**4**	—	17.0 ± 2.0	56.7 ± 0.6
**5**	—	13.4 ± 0.1	14.5 ± 2.1
**6**	—	23.4 ± 0.2	40.6 ± 1.6
**7**	—	18.2 ± 1.4	12.37 ± 2.1
**8**	—	14.9 ± 1.5	27.9 ± 0.4

*Note:* — = inactive.

**FIGURE 3:**
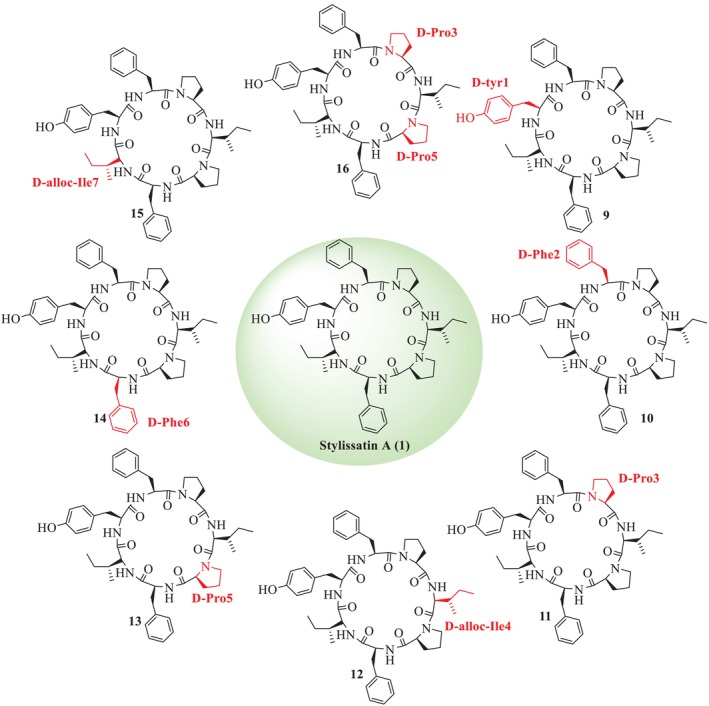
SPPS of analogues of SA (**1**) by Zhang et al. [36].

**FIGURE 4:**
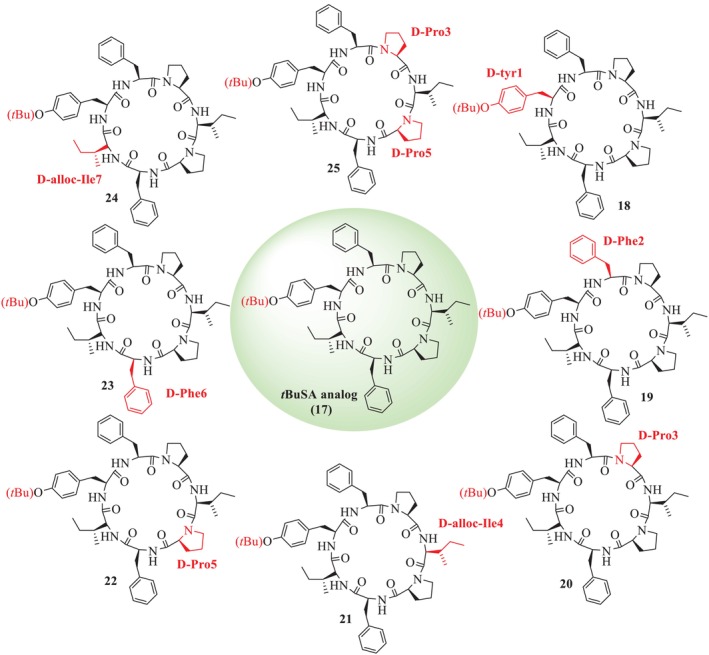
SPPS of analogues of SA (**1**) by Zhang et al. [36].

Zhang et al. [36] also reported the total synthesis of SA (**1**) and its analogues (**9–16**, **18–25**) through SP by replacing L with D amino acid residues to SA [36]. Further, the Tyr hydroxyl group of SA was substituted by *tert‐*butyl ether (*t*Bu) to synthesize *t*BuSA analogue (**17**), along with the alteration of L‐Ile to D‐allo‐Ile^4^‐*t*‐BuSA (**21**), as illustrated in Figures 3 and 4. Among the other eight SA analogues (**9–16**), D‐Tyr^1^‐SA (**2**) was found to show a higher anti‐inflammatory activity (EC_50_ = 60 μM) than (**1**), with reasonable cytotoxicity. In addition, among the *t*BuSA analogues (**18–25**), D‐Tyr^1^‐*t*BuSA, D‐allo‐Ile^7^‐*t*BuSA, and D‐Pro^3^‐*t*BuSA (compounds **8**, **24**, and **25**) effectively inhibited nitric oxide (NO) production, with EC_50_ values of 12, 20, and 17 μM, respectively, and exhibited low cytotoxicity. These analogues demonstrated stronger anti‐inflammatory activity compared to the standard drug indomethacin (EC_50_ = 50 μM). Other *t*BuSA analogues, including D‐Phe^2^‐tBuSA, D‐Pro^3^‐*t*BuSA, D‐Pro^5^‐*t*BuSA, and D‐Phe^6^‐*t*BuSA (compounds **19**, **20**, **22**, and **23**), showed EC_50_ values comparable to that of compound **17**. The respective activities are summarized in Table 2. Furthermore, they also described that both SA (**1**) and *t*BuSA (**17**) inhibited the differentiation of murine 3T3‐L1 preadipocytes (EC_50_ = 9.1 and 1.9 μM) (Table 3). Seven *t*BuSA analogues with D‐amino acid, except for (**20**), were also effective at 10 μM, and notably, D‐Tyr^1^‐*t*BuSA (**18**) had the most potent inhibitory effect (EC_50_ = 440 nM). The adipocyte differentiation‐inhibitory activities of these SA analogues were more potent as compared to the standard hydrocortisone (EC_50_ = 155 μM). The *t*BuSA (**17**) and its D‐Tyr^1^ analogue (**18**) were reported to show a 3.3‐ to 4.3‐fold higher inhibitory profile toward triglyceride accumulation with respect to the natural product and its D‐Tyr^1^ analogue (**1**), respectively. Furthermore, it was reported that SA (**1**), *t*BuSA (**17**), and its D‐Tyr^1^ isomer (**18**) potently reduced triglyceride accumulation in mature adipocytes after 4 days of treatment (EC_50_ = 6.1, 1.9, and 7.0 μM, respectively), given in Table 4.

**TABLE 2:** Inhibition of NO production by SA analogues after 24 h in murine macrophage RAW264.7 cells.

Analogues	NO inhibition (EC_50_ μM)
**SA** (**1**)	73
**9**	60
**10**	> 200
**11**	> 200
**12**	140
**13**	> 200
**14**	> 200
**15**	> 200
**16**	> 200

**TABLE 3:** Inhibition of NO production by SA analogues after 24 h in murine macrophage RAW264.7 cells.

Analogues	NO inhibition (EC_50_ μM)
***t*BuSA** (**17**)	73
**18**	12
**19**	11
**20**	15
**21**	13
**22**	3
**23**	19
**24**	20
**25**	17

**TABLE 4:** Inhibition of differentiation of murine preadipocytes (3T3‐L1) by SA and its analogues.

Analogues	EC_50_ (μM)
**SA** (**1**)	9.1
**17**	2.8
**16**	1.9
**18**	0.44
**19**	4.7
**20**	27
**21**	6.8
**22**	4.2
**23**	4.3
**24**	5.7
**25**	5.0

**FIGURE 5:**
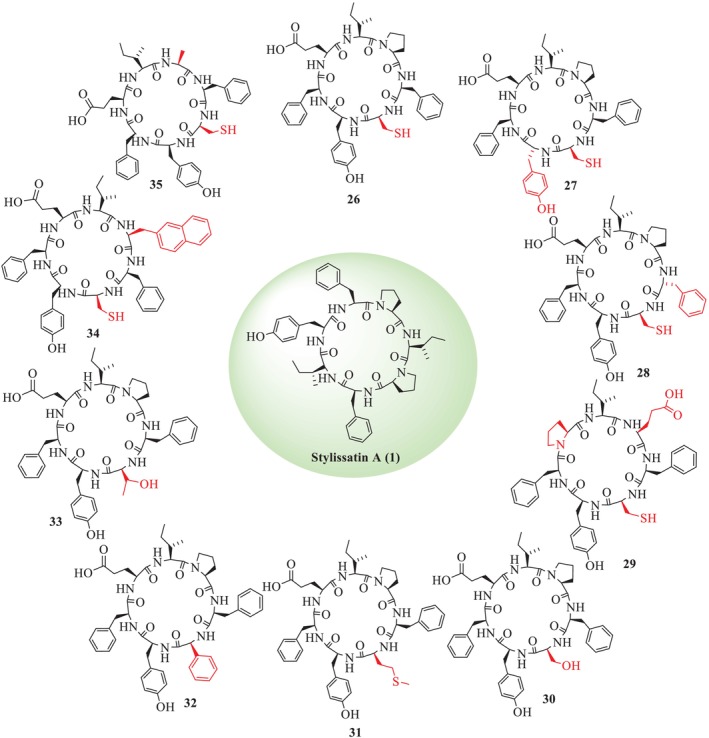
SPPS of analogues of SA (**1**) reported by Aaqib et al. [25].

Aaqib et al. also reported the analogues of SA (**26–35**) by substitution of amino acid residues at positions 2, 4, and 6 by unusual amino acids, along with exchanging the position of residues in the same sequence, given in Figure 5 [25]. The crude analogues were purified through reverse phase HPLC by using an isocratic system having a mobile phase, that is, 40% acetonitrile and 60% water with 0.08% TFA. The structures of all analogues were confirmed through various 1D (^1^H‐NMR) and 2D (HSQC, HMBC, COSY and NOESY) NMR techniques. HMBC was carried out to confirm the correlation of *β* protons with *α* and *δ* protons with *β* protons of each amino acid. The *trans* isomerism of the Pro‐amide bond of all analogues was confirmed through ^13^C‐NMR (DEPT‐135°), which revealed that the chemical shift difference of C*β*−C*γ* of Pro^4^ is 4 to 6 ppm. The amino acid sequence of each analogue was also confirmed using NOESY correlations between the amide (‐NH) protons of neighboring amino acids. In addition, they also reported that the *α* CH proton of Phe^7^ exhibited a strong correlation with *δ* protons of Pro^6^, which further supported the *trans* isomerism of analogues. The amide ‐NH proton of each amino acid was determined from ^1^H‐^1^H COSY correlation with the *α*‐methine proton (‐*α*CH) of the same amino acid residue.

These analogues were evaluated for their effect on NO production, T‐cell proliferation, IL‐2, and ROS inhibition. None of these analogues were found to be active towards IL‐2, T‐cell proliferation, and ROS inhibition. Surprisingly, the analogue (**26**) with Cys at position 2 showed an excellent effect on NO production (IC_50_ = 46.0 ± 2.2 μM), as compared to the natural product SA, shown in Table 5.

**TABLE 5:** NO inhibition activity of analogues of SA.

Analogues	NO inhibition (μM)	Inhibition of TNF‐α, IL‐1β, caspase‐1, and ASC (μM)
**SA** (**1**)	87	—
**26**	46.0 ± 2.2	25
**27**	59.1 ± 2.5	—
**28**	74.3 ± 4.0	—
**29**	> 100	—
**30**	86.4 ± 6.1	—
**31**	87.1 ± 0.1	—
**32**	> 100	—
**33**	> 100	—
**34**	85.0 ± 4.0	—
**35**	85.0 ± 4.0	—

*Note:* — = inactive.

**FIGURE 6:**
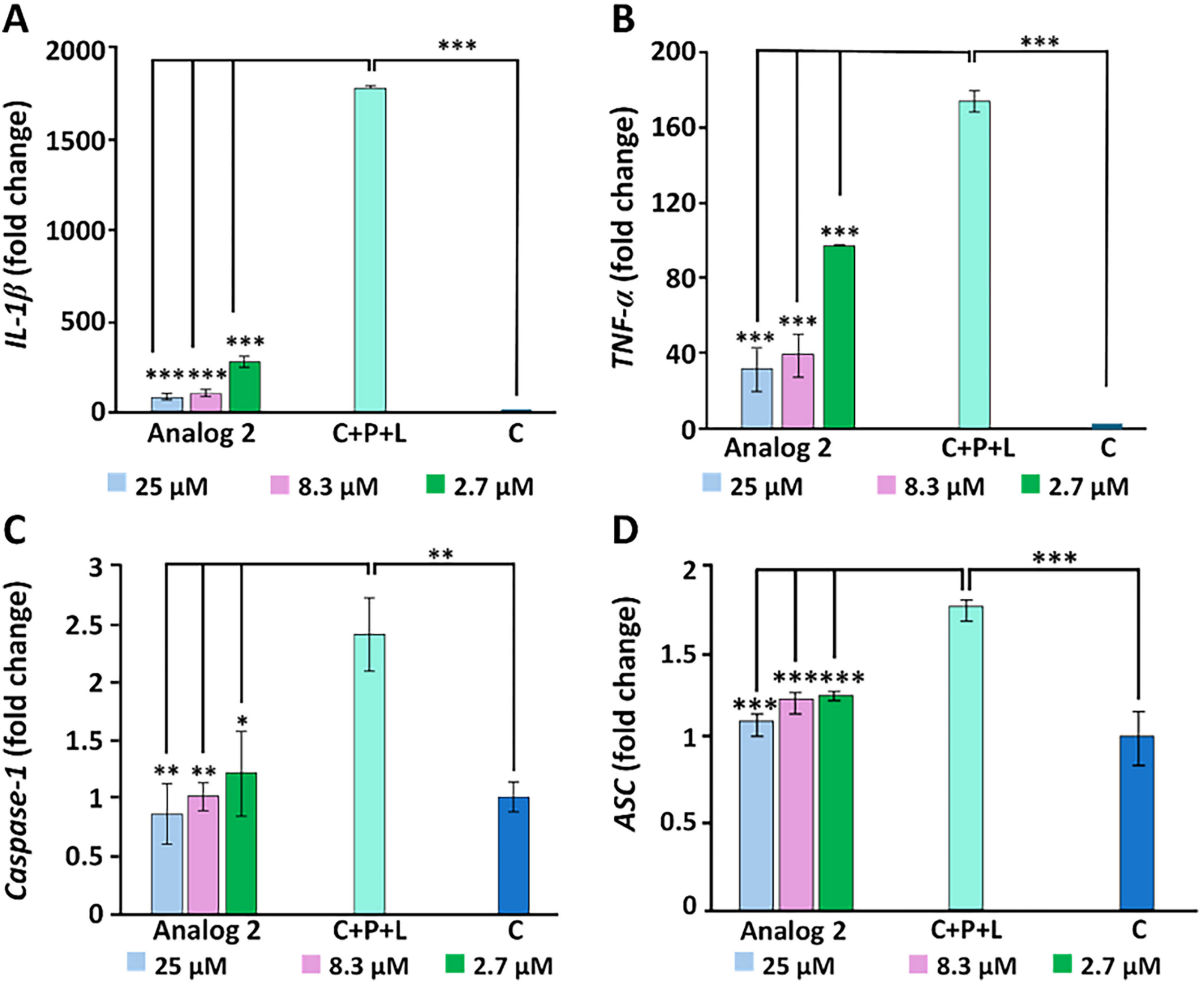
The suppression outcome of analogue **27** on pro‐inflammatory molecules in THP‐1 cells, where C = untreated cells, C + P + L = LPS activated cells primed with PMA. Results were accurately calculated by one‐way ANOVA. ***p ≤ 0.001, **p ≤ 0.005, *p ≤ 0.05. This figure is blurred; it needs to be of higher resolution.

The analogue **26** also suppressed the production of pro‐inflammatory mediators, TNF‐*α*, interleukin‐1*β*, caspase‐1, and ASC (Figure 6), which further supports its anti‐inflammatory and likely therapeutic effects. In addition, it was found to be noncytotoxic to normal mammalian origin cell lines. All other analogues were also found to show good activity as compared to the natural product in Table 5.

**FIGURE 7:**
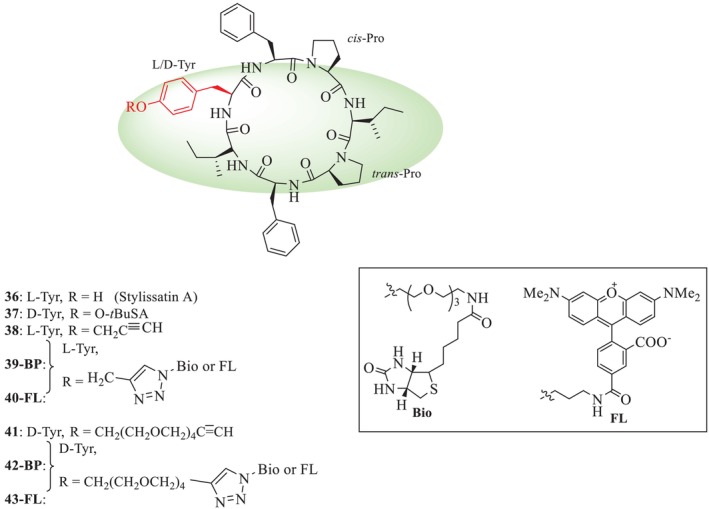
Synthesis of analogues of SA (**1**) by Kita et al. [37].

Kita et al. recently reported the analogues of SA and their inhibition towards membranous neuraminidase 1 function by interacting with lysosomal protective protein cathepsin A (PPCA) [37]. Among these synthetic SA analogues (SAs), a *tert*‐butyl ether analogue, D‐Tyr^1^‐O‐*t*BuSA (**37**), remarkably inhibited nitric oxide (NO) production with little cytotoxicity. Analogue (**37**) also inhibited the production of the pro‐inflammatory cytokines IL‐6 and TNF‐*α* and suppressed the production of *i*NOS. SA biotin probe (**3‐BP**) prepared from propargyl SA (**37**), acyl‐CoA dehydrogenase long chain (ACADL) was identified as a target protein from macrophage lysates. All analogues are given in Figure 7. The D‐Tyr^1^ analogue (**37**) showed the highest inhibitory potential on fat accumulation in preadipocytes (EC_50_ = 0.44 μM) with little cytotoxicity (IC_50_ > 200 μM). In addition, **36**, **37**, and their L‐Tyr^1^ isomer *t*‐BuSA potently reduced triglyceride accumulation in mature adipocytes (EC_50_ = 6.1, 7.0, and 1.9 μM, respectively).

Compound (**41**) pinpointed moderate anti‐inflammatory activity against LPS‐treated RAW264.7 macrophages (EC_50_ = 40 μM), similar to (**36**) and D‐Tyr^1^‐SA (**37**) (EC_50_ = 73 and 60 μM). For comparison, an L‐SA fluorescent probe (**3‐FL**) that lacks an extended PEG4 linker was synthesized from propargyl SA (**38**). Both (**40‐FL**) and (**43‐FL**) were reported to enter the cell relatively rapidly within 30 min and stained specific organelles for over 6 h.

Further, the same authors reported through molecular docking studies of these analogues with lysosomal protective protein cathepsin A (PPCA) binding affinity. The results revealed that the natural product **36** and its analogue **37** interacted with activated PPCA at the Neu1 binding site (with 1.31 and 0.89 kcal/mol, respectively). Thus, this recorded inhibitory capacity might serve as a basis towards the development of novel peptide‐based drugs in the future.

Based on the discussion above, several structure–activity relationships (SARs) can be identified regarding the role of specific amino acid residues in the natural product SA and its ability to reduce NO production. Notably, protection of the tyrosine hydroxyl group using tert‐butyl, propargyl, methyl, or benzyl groups (forming corresponding ethers) was found to enhance the NO‐reducing activity of SA derivatives. Additionally, the stereochemistry of tyrosine (D‐ and L‐forms), along with modifications on its hydroxyl group, was shown to be crucial not only for inhibiting NO production but also for suppressing the differentiation of murine 3T3‐L1 preadipocytes, highlighting a potential anti‐obesity effect. Furthermore, the position and identity of specific amino acids within the peptide sequence significantly influenced bioactivity. For example, substitutions at Pro^6^ and Ile^2^ specifically with Glu, Ala, or Cys altered biological activity, suggesting that the nature and positioning of these residues are critical determinants of the peptide's bioactivity. These SAR insights underscore the importance of precise residue selection and chemical modification for optimizing the therapeutic potential of SA derivatives.

## 4 Conclusion

This mini‐review underscores the significance of natural cyclic peptides derived from marine sponges, with a particular focus on stylissatins A–D. Among these, stylissatin A (SA) emerges as especially noteworthy due to its promising biological activities. Extensive efforts have been made by various research groups to synthesize SA through both solid and solution‐phase total synthesis approaches. In addition to the native compound, a wide range of SA analogues have been developed and biologically evaluated. Findings suggest that specific amino acid residues such as Ile at position 2, Pro at positions 4 and 6, and Tyr at position 1 are key contributors to the peptide's therapeutic potential. The novelty and added value of this work lie in its comprehensive and critical analysis of the synthetic strategies for SA, emphasizing recent advances, current challenges, and future opportunities. By systematically comparing existing methodologies in terms of efficiency, yield, and sustainability, this review serves as a valuable resource for researchers seeking to optimize synthetic routes or develop novel SA‐based analogues with enhanced therapeutic potential, including anti‐inflammatory, anticancer, and anti‐obesity activities.

## 5 Future Perspective

Further optimization is essential to develop more potent analogues of the natural cyclic peptide stylissatin A (SA). One promising strategy involves substituting the Ile at position 2 and the Pro at position 4 with unnatural amino acids to improve biological activity and selectivity. Additionally, modifying the hydroxyl group of tyrosine by introducing alkyl or acyl chains, either hydrophilic or hydrophobic, could enhance membrane permeability and receptor binding. Incorporating N‐methylated amino acid residues may also improve metabolic stability and lead to the development of novel anti‐inflammatory agents. Beyond conventional screening, evaluating these analogues in enzyme inhibition assays may reveal new mechanistic pathways and therapeutic applications. Furthermore, investigating their ability to modulate immune checkpoint proteins, such as PD‐1 and PD‐L1, may provide insights into their potential as T‐cell activation regulators, offering a new direction in peptide‐based immunotherapy. Extending similar synthetic optimization approaches to stylissatin B–D could also contribute to the discovery of potent peptide‐based anticancer candidates. Emphasis could be given to ligand observed NMR methods [38] that have considerably expanded the potentialities for NMR investigation of protein‐ligand interactions. The use of STD experiments, transfer NOEs for pharmacophore mapping (INPHARMA) NMR in combination with computational methods [37, 38, 39, 40] can provide excellent means for the investigation of conformational changes of peptide ligand at the receptor site.

## Conflicts of Interest

The authors declare no conflicts of interest.

## Data Availability

The data that support the findings of this study are available from the corresponding author upon reasonable request.
